# IL18BP in cancer: a double-edged sword and emerging therapeutic opportunity

**DOI:** 10.3389/fimmu.2026.1922089

**Published:** 2026-10-07

**Authors:** Chenshen Huang, Yiqing Jiang, Ning Wang, Bing Du, Qi Huang

**Affiliations:** 1 Fuzhou University Affiliated Provincial Hospital, School of Medicine, Fuzhou University, Fuzhou, China; 2 Huzhou Central Hospital, The Fifth School of Clinical Medicine of Zhejiang Chinese Medical University, Huzhou, China; 3 Shanghai Frontiers Science Center of Genome Editing and Cell Therapy, Shanghai Key Laboratory of Regulatory Biology, Institute of Biomedical Sciences and School of Life Sciences, East China Normal University, Shanghai, China; 4 Shanghai Tongji Hospital, School of Medicine, Tongji University, Shanghai, China

**Keywords:** biomarkers, cancer immunotherapy, cytokine, Interleukin-18-binding protein (IL18BP), immune checkpoint

## Abstract

Interleukin-18-binding protein (IL18BP) is a pivotal extracellular gatekeeper of IL-18 activity and an emerging therapeutic target in cancer. By sequestering mature IL-18, IL18BP controls the receptor-accessible free ligand within the tumor microenvironment. The consequences are highly context dependent: IL18BP may suppress beneficial natural killer- and T-cell responses, yet also restrain IL-18-driven immune tolerance, inflammation, and metastatic adaptation. Here, we outline the mechanisms, cellular sources, tissue distribution, and cancer-specific roles of the IL-18/IL18BP axis. We emphasize that total, bound, and free IL-18 are biologically distinct and must be interpreted alongside IL18BP abundance, receptor competence, responder-cell identity, and treatment context. Accordingly, IL18BP blockade or decoy-resistant IL-18 agonists may restore antitumor immunity in selected settings, whereas preserving or enhancing IL-18 neutralization may be protective in others. Precision therapeutic tuning, rather than indiscriminate blockade, will be essential to translate this axis into effective biomarkers and cancer treatments.

## 1 Introduction

Tumor-associated inflammation can support immune surveillance or drive immune escape, tissue remodeling, and treatment resistance. Interleukin-18 (IL-18) lies at this intersection because it links innate inflammatory sensing to interferon-gamma (IFN-γ)-producing lymphocytes and myeloid regulation. In permissive contexts, mature IL-18 can amplify the activity of natural killer (NK) cells and T cells; in others, IL-18 signaling is associated with suppressive immune circuits, metastatic-niche biology, or inflammatory risk (1–3). Thus, neither IL-18 abundance nor a generic pro- or antitumor label adequately describes the pathway.

The relevant biological unit is the IL-18/interleukin-18-binding protein (IL18BP) axis. IL18BP is a soluble protein encoded independently of IL-18 receptor chains that sequesters extracellular IL-18 and reduces the receptor-competent free-ligand pool (4–6). IFN-γ-linked feedback can induce IL18BP in selected epithelial or tumor-compatible systems (7, 8), so immune activation may also generate a soluble constraint on subsequent IL-18 activity. Functional interpretation therefore requires the IL-18 source, maturation and binding state, IL18BP source, receptor competence, responding cell type, and tissue compartment to be considered together.

Cancer studies illustrate why IL18BP can have opposing therapeutic implications. In some preclinical settings, anti-IL18BP blockade or IL18BP-resistant ligands reveal IL18BP as a checkpoint-like barrier to antitumor immunity (9–11). In pancreatic and hepatic-metastasis models, however, IL-18 can support tolerance, endothelial adhesion, or metastatic biology, leaving IL18BP-mediated neutralization potentially protective (12–14).

This review examines these opposing roles of IL18BP. We first define how IL-18 production, maturation, release, and sequestration determine extracellular IL-18 availability, and how receptor competence shapes cellular responses. We then examine IL18BP sources and regulation in the tumor microenvironment (TME), compare direct-source, biomarker, transcriptomic, and interventional evidence across cancers, and integrate the antitumor and tumor-supportive outcomes of IL-18 signaling. Finally, we evaluate biomarker and therapeutic strategies according to axis state, localization, responding cells, and inflammatory safety. The goal is to identify when IL18BP is a therapeutic barrier, when it is a protective brake, and which evidence is still needed for precision tuning of the axis. **Figure 1** summarizes extracellular IL-18 availability, IL18BP sequestration, sequential receptor assembly and IFN-γ-linked feedback. **Figure 2** presents context-specific tumor responses and five distinct intervention strategies.

**Figure 1:**
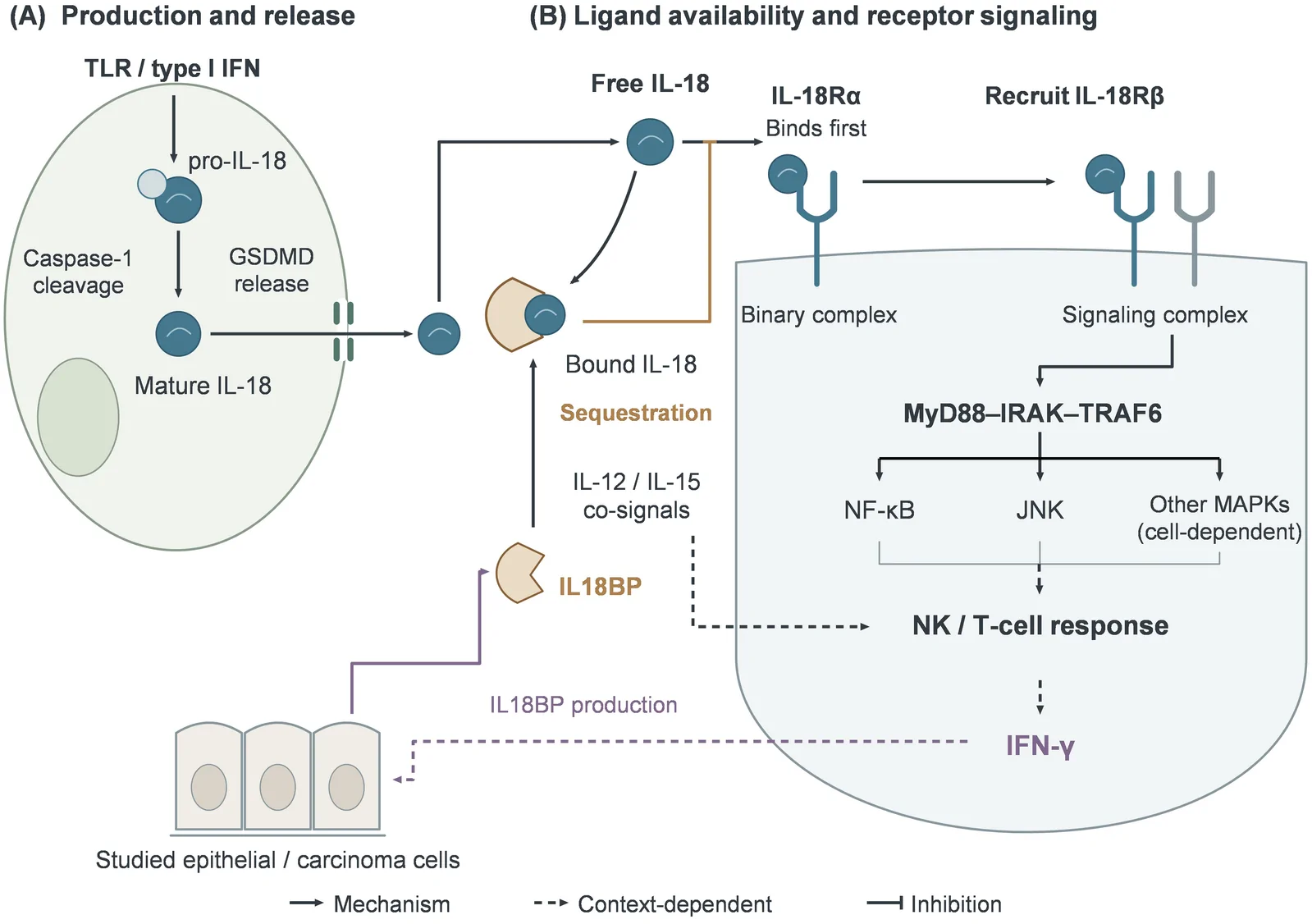
IL-18 availability, receptor signaling and IL18BP feedback. **(A)** Production and release. Precursor abundance, proteolytic maturation and extracellular release are separate controls. Representative TLR/type I interferon inputs refer to mouse bone marrow-derived macrophages and primary human monocytes. Caspase-1-linked maturation generates receptor-binding surfaces. GSDMD-dependent IL-18 release can occur without complete cell lysis; release depends on stimulus and cell state. The extracellular mature pool supplies the free ligand shown in **(B)** Ligand availability and receptor signaling. IL18BP binding limits ligand access to receptors. Free mature IL-18 first binds IL-18Rα the binary complex then recruits IL-18Rβ. The MyD88-IRAK-TRAF6 proximal module feeds separable NF-κB, JNK and additional cell-dependent MAPK outputs. In permissive NK- or T-cell contexts, IL-18 contributes to IFN-γ production. IFN-γ induces IL18BP expression and release in studied epithelial and carcinoma-cell systems. The feedback path combines these producer-cell findings with lymphocyte responses and is context dependent.

**Figure 2:**
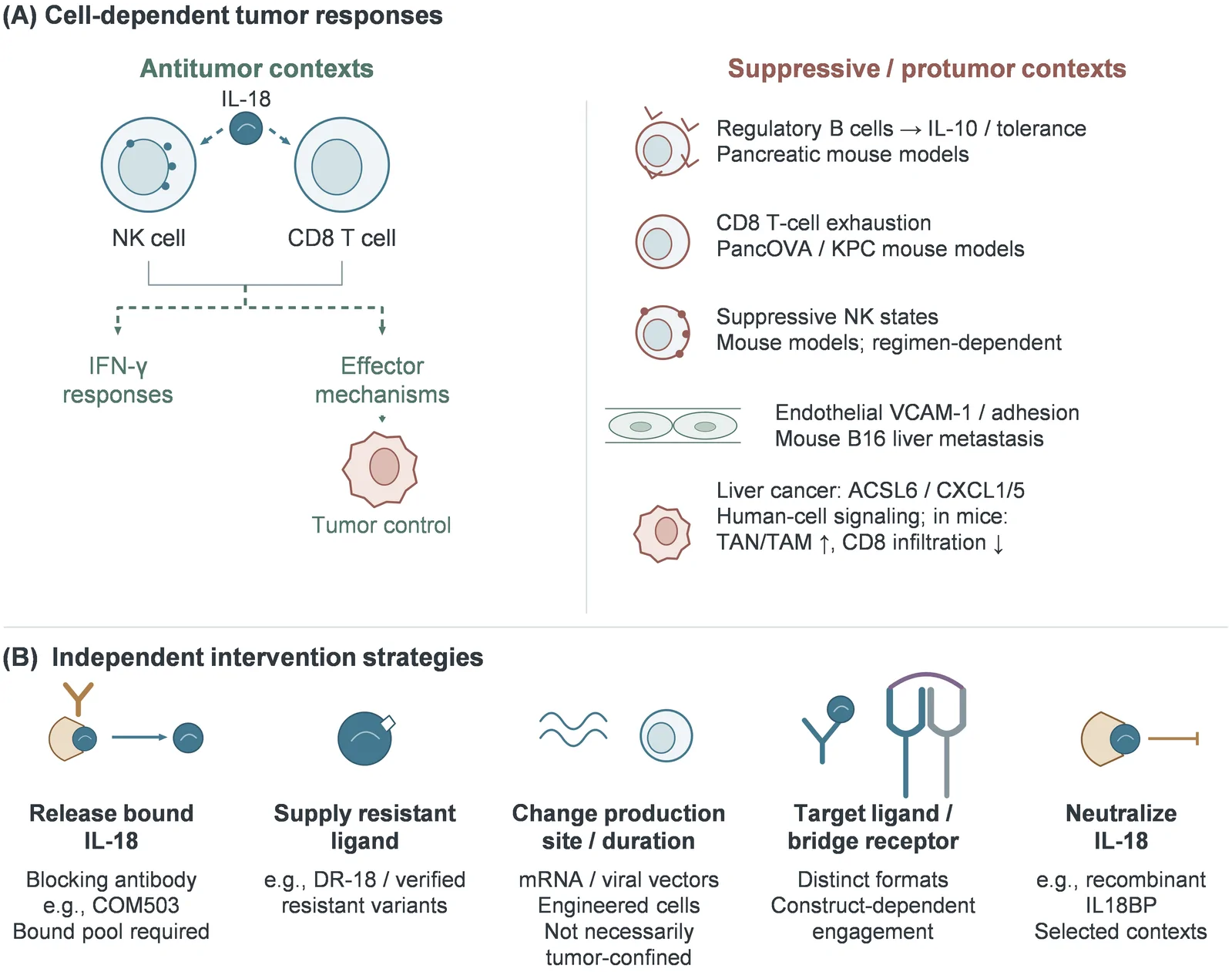
Cell-dependent tumor responses and distinct intervention strategies. **(A)** Cell-dependent tumor responses. IFN-γ and effector responses are parallel; NK/CD8 T-cell and cytokine requirements vary by model and regimen. **(B)** Five independent strategies. (1) Release bound IL-18 with IL18BP-blocking antibodies such as COM503, requiring a recoverable bound pool. (2) Supply DR-18 or verified IL18BP-resistant variants. (3) Alter production site or duration with mRNA, vectors or engineered cells, without ensuring tumor-confined exposure or IL18BP resistance. (4) Use target-directed IL-18 constructs or receptor-bridging agonists. (5) Neutralize IL-18, including with recombinant IL18BP, in selected suppressive or metastatic models.

## 2 Biology of the IL-18/IL18BP axis

The IL-18/IL18BP axis controls the amount of receptor-competent free IL-18 available in tissue. IL-18 can support IFN-γ-linked antitumor immunity, but its biological effect depends on how much mature cytokine is generated, whether it remains unbound, which cells express the IL-18 receptor complex, and whether IL18BP is present at concentrations sufficient to sequester the ligand before receptor engagement (5, 6). This matters in cancer because tumors can contain IL-18, IL18BP, receptor-bearing lymphocytes, inflammatory myeloid cells, and therapy-induced cytokine programs in the same tissue compartment. Total IL-18 abundance must therefore be separated from the unbound and more functionally relevant pool of free IL-18 that can reach IL-18-responsive cells (1, 15).

### 2.1 IL-18 production, maturation, and release

IL-18 availability is controlled at three distinct levels: expression or pre-existing abundance of pro-IL-18, proteolytic maturation, and extracellular release. A precursor pool can be present before stimulation in some myeloid cells, so IL-18 does not always follow the inducible-precursor model typical of interleukin-1 beta. Expression can nevertheless increase in defined contexts. In murine bone marrow-derived macrophages, Toll-like receptor (TLR) stimulation increased *Il18* messenger RNA (mRNA) and pro-IL-18 protein through a type I interferon-dependent program; in primary human monocytes, TLR and type I interferon signals cooperated to induce and sustain *IL18* expression (16, 17). In these defined myeloid systems, pattern-recognition and type I interferon signaling therefore act as representative upstream inputs.

Precursor abundance is distinct from cytokine maturation. Inflammasome-associated caspase-1 cleaves pro-IL-18, and structural analysis shows that cleavage triggers conformational rearrangements that form the binding surfaces required for IL-18 receptor and IL18BP engagement (18). Increased *IL18* transcription or pro-IL-18 protein is therefore insufficient evidence that extracellular, receptor-competent cytokine has been generated.

Release is a third control point. Primary macrophage studies show that IL-18 can exit through gasdermin D-dependent membrane permeabilization without complete cell lysis, whereas lytic membrane rupture can also release cytosolic cytokine (19, 20). The contribution of each route is stimulus- and cell-state-dependent. Only extracellular mature IL-18 can be sequestered by IL18BP or engage IL-18 receptors, so measurements of expression, cleavage, and release capture different stages of the axis.

### 2.2 IL-18 receptor signaling and receptor competence

Free mature IL-18 first binds IL-18 receptor alpha (IL-18Rα encoded by *IL18R1*) to form a binary complex, which then recruits IL-18 receptor beta (IL-18Rβ encoded by *IL18RAP*) and completes the signaling-competent ternary receptor complex (21). Receptor assembly is therefore sequential rather than a set of parallel binding events. Formation of the ternary complex is followed by Toll/interleukin-1 receptor (TIR) domain signaling that requires myeloid differentiation primary response 88 (MyD88); interleukin-1 receptor-associated kinase (IRAK) is recruited and activated at the receptor complex and associates with tumor necrosis factor receptor-associated factor 6 (TRAF6) (22–24). This MyD88-IRAK-TRAF6 module is receptor proximal and feeds downstream branches rather than extending as one serial chain. Primary loss-of-function studies support nuclear factor kappa B (NF-κB) and c-Jun N-terminal kinase (JNK) as separable downstream outputs, whereas p38 and extracellular signal-regulated kinase (ERK) contributions have been demonstrated in selected NK-cell models (22, 25, 26). Interleukin-12 (IL-12) and interleukin-15 (IL-15) are contextual co-signals that alter responder-cell competence or effector output; they are not components of the IL-18R-proximal cascade (27, 28).

IL-18’s immune-activating biology makes the cytokine attractive for cancer immunotherapy: if receptor-competent free IL-18 reaches appropriately primed lymphocytes, it can amplify inflammatory and cytotoxic programs. Receptor competence, however, is more than receptor expression. *IL18R1* or *IL18RAP* transcripts in a tumor do not necessarily indicate that IL-18 agonism will provide therapeutic benefit. The responding cell type, activation state, tissue niche, and checkpoint-therapy background all shape the direction of IL-18 biology. IL-18R signaling alone is insufficient to indicate useful antitumor activity. Decoy-resistant strategies aim to preserve IL-18 receptor activation while avoiding sequestration by IL18BP (10, 11).

### 2.3 IL18BP discovery, isoforms, and sequestration of IL-18

IL18BP was purified from human urine and identified as a secreted immunoglobulin (Ig)-superfamily protein rather than a canonical IL-18 receptor chain. The discovery study found no transmembrane-domain exon, distinguishing IL18BP from a shed form of IL-18R and from IL18R1 or IL18RAP (4). Structural interpretation requires a construct boundary: the full-length recombinant human protein examined by Detry et al. was heavily and heterogeneously glycosylated, whereas the IL-18-bound crystal structure used a bioactive N-terminally truncated, glycan-trimmed construct. Its resolved beta-sandwich core binds an IL-18 surface overlapping the IL-18Rα-binding-epitope, explaining extracellular sequestration and competition with productive receptor assembly (6). These data define the binding interface of the engineered construct but leave the structure and physiological oligomeric state of the four full-length splice variants unresolved.

Using the a-d nomenclature of the original functional study, the four human IL18BP splice isoforms share an N-terminal mature sequence but acquire different carboxyl termini. IL18BPa and IL18BPc retain a complete Ig domain, but their terminal 29 amino acids differ. In the tested binding assay, IL18BPa had the highest affinity for mature human IL-18, with a reported dissociation constant (Kd) of 0.399 +/- 0.034 nM, whereas IL18BPc bound with lower affinity (2.94 +/- 0.86 nM); both strongly neutralized IL-18 at a two-fold molar excess. In IL18BPb and IL18BPd, in-exon splicing of exon 4 produces frameshifts and new carboxyl-terminal sequences while disrupting the complete Ig domain; neither isoform bound or neutralized IL-18 in the tested assays (5). The functional differences therefore extend beyond simple retention of a complete Ig domain. The functional comparison does not establish whether the distinct carboxyl termini cause the affinity difference between IL18BPa and IL18BPc.

The IFN-γ induction results discussed below establish increased IL18BP expression and release, but do not by themselves identify a mechanism of splice-isoform selection (7, 8). Cancer isoform evidence is limited: a prostate study inferred predominant IL18BPa in PC3 and DU145 cells from a single approximately 42-kDa immunoblot band, while a pancreatic carcinoma study specifically assessed IL18BPa (29, 30). These findings should not be read as a four-isoform comparison of secretion or neutralization across patient tumors. For the measurements discussed here, gene-level *IL18BP* RNA or total protein alone cannot establish isoform-specific secretion or neutralizing activity in patient tumors.

### 2.4 IFN-γ-induced IL18BP feedback

IL18BP production has constitutive and inducible components. In permissive NK- or T-cell contexts, IL-18 receptor signaling can contribute to IFN-γ production (22, 26, 27). In epithelial/carcinoma-compatible systems, IFN-γ can then induce IL18BPa expression and release. Paulukat et al. demonstrated this response in DLD-1 cells and additional colon carcinoma/epithelial systems; the induced activity reduced IFN-γ release from IL-12/IL-18-stimulated peripheral blood mononuclear cells (PBMCs) (7). The resulting feedback can limit subsequent IL-18 bioactivity, but its operation depends on cellular context.

In Hep G2 cells, Hurgin et al. showed that IFN-γ-induced *IL18BP* transcription depended on *de novo* protein synthesis and on a gamma-activated sequence (GAS) and an interferon regulatory factor 1 response element (IRF-E) in the promoter. DNA-binding and co-immunoprecipitation experiments identified an IFN-γ-induced complex of interferon regulatory factor 1 (IRF-1) and CCAAT/enhancer-binding protein beta (C/EBPbeta), with binding to probes from the proximal promoter (8). These promoter experiments support an active transcriptional response to IFN-γ.

Tumor studies indicate that this feedback is context dependent. IL18BP was inducible in ovarian cancer systems (31). In prostate cancer, responsive and nonresponsive cell lines differed in IFN-γ-responsive IL18BPa secretion alongside lower Janus kinase 1 (JAK1) expression and trace or absent IFN-γ-induced signal transducer and activator of transcription 1 (STAT1) phosphorylation in the nonresponsive lines (29). Thus, an inflamed tumor may generate both immune-activating cytokines and a soluble brake on IL-18, but whether this brake is induced and which cells provide it remain model dependent.

### 2.5 Total, free, and bound IL-18 as distinct readouts

Total IL-18, IL18BP, the IL-18/IL18BP complex, free bioactive IL-18, and receptor competence are distinct measurement domains. Total IL-18 is not equivalent to receptor-competent free ligand, and functional neutralization depends on ligand binding state, assay design, and the presence of nearby responsive cells rather than IL18BP abundance alone (32). **Table 1** summarizes these readouts and their interpretation limits.

**Table 1:** Research readouts and interpretation limits for the IL-18/IL18BP axis.

Readout	Information provided	Interpretation and reporting limits
Total IL-18	Broad ligand abundance in a specified tissue, plasma, or other sample type (1, 15).	It does not distinguish IL18BP-bound, otherwise inactive, or receptor-accessible free ligand. Report sample type and assay; do not use total IL-18 as a proxy for bioactivity.
IL18BP abundance	Soluble-antagonist abundance and a possible tumor, biofluid, or cellular-state signal (29, 31, 33).	Ligand occupancy, secreted functional neutralization, cellular source, and targetability require additional measurements. Interpret with ligand compartments, source mapping, and responder cells.
IL-18/IL18BP complex or another bound/inactive-ligand readout	The IL-18/IL18BP complex assay identifies a bound compartment; other inactive-ligand readouts require their own assay-specific interpretation. The complex assay has been used in metastatic non-small-cell lung cancer samples (33).	It does not show that useful responder cells are present or that releasing the ligand would be beneficial.
Directly measured free IL-18	Assay-defined unbound ligand; whether the assay also establishes mature or bioactive IL-18 depends on its detection design (32, 54).	Report the assay, sample compartment, and specificity for free, mature, or bioactive ligand. The measurement alone does not establish an oncology cutoff.
Calculated free IL-18	Calculated free IL-18 is an estimate from measured analytes and binding assumptions, not a direct assay (33).	Report inputs, binding model, and assumptions; the estimate alone does not establish an oncology cutoff.
*IL18R1*/*IL18RAP* expression and responder mapping	Potential receptor-bearing cells or compartments (1, 11, 35).	Expression alone does not prove signaling competence or predict antitumor, suppressive, or inflammatory direction. Combine with cell identity/state, ligand availability, tumor lineage, and treatment context.
Tissue or cellular source mapping	Localization of IL-18 or IL18BP signals to tumor, epithelial, leukocyte, or myeloid compartments (29, 31, 34, 48, 49).	Source alone does not quantify free-ligand activity or establish causal immune suppression. Report whether evidence is transcript, protein, spatial, single-cell, or functional; treat methods as complementary.
Therapy-linked longitudinal sampling	Within-patient change in ligand partitioning, IL18BP abundance, responder context, or inflammatory signals (11, 33, 51, 54).	Prospective validation is required before longitudinal changes can guide response, toxicity, or treatment selection. Use for prospective pharmacodynamic and safety research, not current standard-of-care decisions.

IL-18, interleukin-18; IL18BP, interleukin-18-binding protein; *IL18R1*, IL-18 receptor alpha-chain gene; *IL18RAP*, IL-18 receptor accessory-protein/beta-chain gene.

Free IL-18 may be measured directly or calculated from measured analytes and binding assumptions; these readouts should be distinguished (32, 33). The metastatic non-small-cell lung cancer (NSCLC) example is discussed in Section 7.

Tissue expression ratios can add source and context information but should not be relabeled as free IL-18 or bioactivity; this distinction applies to the ovarian IL-18/IL18BP ratio reported by Medina et al. (34). No single readout resolves the axis, so ligand state, IL18BP abundance, source, and receptor competence should be reported separately.

The axis therefore centers on extracellular ligand availability, receptor competence, and tissue context. IL18BP can be a barrier to useful immunity or a protective brake, but that direction cannot be inferred from any single analyte.

## 3 IL18BP in the tumor microenvironment

The tumor microenvironment adds a spatial and cellular layer to the IL-18/IL18BP axis. In tumors, IL-18, IL18BP, IL-18R-bearing cells, and inflammatory feedback signals may be distributed across malignant epithelial cells, tumor-associated leukocytes, monocyte-derived macrophages, stromal or epithelial niches, ascites, plasma, and therapy-altered immune infiltrates. IL18BP is therefore most informative when its cellular source, tissue compartment, inflammatory regulation, and nearby IL-18-responsive cells are considered together. In one tumor state, local IL18BP may limit useful IL-18-driven lymphocyte activity; in another, it may restrain harmful inflammation (3).

### 3.1 Tumor-associated IL18BP sources

Epithelial ovarian cancer and prostate cancer provide well-described examples of tumor-associated IL18BP production. In epithelial ovarian cancer, Carbotti et al. reported increased IL18BP expression and release in the tumor microenvironment, with evidence from tumor tissue, patient serum and ascites, tumor cells, and tumor-associated leukocyte compartments (31). The ascites signal is especially relevant because it places IL18BP in a soluble tumor-adjacent compartment. These data indicate locally generated IL18BP in ovarian cancer; its functional effect still depends on IL-18 abundance, receptor localization, and cellular context.

Medina et al. compared IL-18, IL18BP, and IL-18 receptor expression in normal and cancerous ovarian tissues and reported a higher IL-18/IL18BP ratio in cancerous tissue (34). Together with Carbotti et al., this shows tumor-site IL18BP production alongside an IL-18-skewed expression profile. The ratio is not a direct measure of free IL-18 or receptor activity.

Prostate cancer provides a second direct source model and illustrates tumor-cell heterogeneity more sharply. Fujita et al. showed that DU145 and PC3 prostate cancer cells expressed and secreted functional IL18BP after IFN-γ stimulation, whereas LNCaP and CWR22 were comparatively nonresponsive in the same experimental setting. The nonresponsive lines had lower JAK1 expression and trace or absent IFN-γ-induced STAT1 phosphorylation (29), connecting tumor-cell responsiveness to the feedback logic described above. In radical-prostatectomy specimens, anti-IL18BP immunohistochemistry stained prostate cancer epithelial cells and cells identified as macrophages within or around inflamed glands (29). However, the tissue study did not report a macrophage lineage/state marker panel, distinguish tissue-resident from recruited macrophages, classify tumor-associated functional states, or measure macrophage-specific IL18BP secretion or neutralizing activity.

A later cross-tumor transcriptomic screen reported higher *IL18BP* RNA in tumor-associated than tissue-resident macrophage populations, but its representative human comparison was lung adenocarcinoma rather than prostate cancer (9). The RNA difference therefore cannot resolve prostate-specific secretion or IL-18-neutralizing activity. Determining whether macrophage ontogeny or functional state alters local IL18BP availability in prostate tumors will require lineage-informed, state-resolved RNA linked to secretion and neutralization assays.

### 3.2 Myeloid states and macrophage–NK-cell circuits

Park et al. examined a macrophage-NK-cell circuit primarily in an orthotopic mouse lung-adenocarcinoma model, with human NSCLC data providing correlative context. In the human tumors, monocyte-derived macrophage frequency was inversely associated with NK-cell frequency; the human cell-frequency and transcript data do not establish patient-tumor IL18BP protein abundance, free IL-18, or IL18BP-specific causality (48).

In the mouse model, tumor-debris uptake promoted a macrophage program governed by triggering receptor expressed on myeloid cells 2 (TREM2). TREM2-high monocyte-derived macrophages contained more *Il18bp* transcript than TREM2-low macrophages or alveolar macrophages, whereas alveolar macrophages were the main *Il18*-transcript-expressing myeloid population (48). In co-culture, *Trem2*-deficient macrophages increased the activation and proliferation markers CD69 and Ki-67 in IL-18/IL-12-stimulated wild-type NK cells, but not in *Il18r1*-deficient NK cells. *In vivo* IL-18 neutralization partially reversed the reduced tumor burden and NK-cell recruitment or activation observed after *Trem2* loss (48). The intervention results point to an IL-18-responsive component of the macrophage-NK circuit while leaving the necessity of IL18BP unresolved.

IL-15 formed a distinct support arm: *Il15* transcript increased in mature dendritic cells enriched in immunoregulatory molecules after *Trem2* loss, and IL-15 neutralization abolished tumor reduction and NK-cell expansion (48). The *Il18bp* transcript comparison and the TREM2, IL-18, and IL-15 interventions associate IL18BP with this circuit without establishing macrophage-derived IL18BP protein abundance or IL18BP-specific necessity. The functional circuit was tested in defined mouse lung-tumor models; its relevance to other TREM2-positive macrophages, M2-like states, tumors, or tissues remains to be determined.

Time-resolved single-cell work in a murine GL261 glioblastoma model placed *Il18bp* within a suppressive monocyte-to-macrophage trajectory (49). This transcript-level trajectory maps source and state over time while leaving secreted IL18BP protein, neutralizing activity, and IL18BP-specific causality unresolved.

### 3.3 Tissue niches and biofluid compartments

Compartment changes the meaning of an IL18BP measurement. Paired ascites/serum data in ovarian cancer support a local soluble compartment; post-digital-rectal-examination urine in prostate cancer is a prostate-enriched biofluid rather than a general blood-biomarker template; and the GL261 mouse glioblastoma trajectory represents changing brain myeloid states (29, 31, 49). These study-internal examples cannot be combined into a cross-tumor baseline, but they show why source, fluid or tissue compartment, and responder-cell proximity should be reported together.

The clinical biofluid data in the Fujita prostate study support biofluid detectability and association with tumor status, without diagnostic validation. Urinary and serum IL18BP differed by tumor-related clinical groupings, whereas serum IL-18 and calculated free IL-18 did not show the same association pattern (29).

A tissue expression ratio, plasma complex concentration, or calculated free-IL-18 estimate answers a different question from local receptor competence. The NSCLC study is evaluated in the Biomarker section; here it shows only that peripheral analytes do not identify the source or responder cells within a tumor (33).

### 3.4 Responder cells and functional interpretation

The functional consequences of IL18BP depend on which receptor-competent cells can access free IL-18. In permissive NK-cell or CD8-positive T-cell contexts, free mature IL-18 can support IFN-γ-linked antitumor activity, motivating attempts to bypass this checkpoint-like barrier with decoy-resistant agonists (10).

Functional interpretation of IL18BP therefore depends on source, extracellular ligand state, receptor-competent responders, tissue compartment, and treatment background. Those features should guide investigation of blockade, bypass, or preservation of IL-18 neutralization; clinical selection will require additional evidence.

## 4 Cancer-type evidence and tissue-specific contexts

Cancer studies address different questions: where IL18BP is produced, how the axis is measured, and what happens when IL-18 signaling is altered. These questions require different evidence and should remain distinct when cancer types are compared. **Table 2** compares selected cancer contexts.

**Table 2:** Evidence and interpretation limits across selected cancer contexts.

Cancer context	Model or sample and main finding	Interpretation and limits
Epithelial ovarian cancer	Human tumor tissue, paired ascites/serum, tumor cells and tumor-associated leukocytes identify IL18BP sources. Ovarian tissue shows an increased IL-18/IL18BP expression ratio; selected ovarian cancer models show interferon-γ- or IL-27-inducible IL18BP (31, 34).	The tissue and fluid comparisons are study-internal, the expression ratio is not a measurement of free IL-18, and these patient observations do not establish the *in vivo* inducing signal.
Prostate cancer	Selected prostate cell lines show interferon-γ-inducible IL18BP and conditioned-medium neutralization; human prostatectomy tissue shows staining, and urine/serum samples establish detectability (29).	Tumor-cell experiments leave macrophage-state-specific secretion unresolved. Clinical biofluid data show association and detectability, not diagnostic performance or cross-cancer comparability.
Gastric adenocarcinoma	Correlative chromatin, transcript, and tissue-staining evidence linking *IL18BP* promoter accessibility and expression with *TP53* and CD4-positive T-cell infiltration (52).	The correlative design leaves source-resolved secretion, free IL-18, functional neutralization, and IL18BP-specific causality unresolved.
Lung cancer/non-small-cell lung cancer	Human NSCLC data show an inverse macrophage-NK-cell frequency association; a mouse lung-tumor model shows TREM2-associated myeloid *Il18bp* transcript (48). A separate metastatic NSCLC cohort treated with immune checkpoint inhibitors measured plasma total IL-18, IL18BPa and IL-18/IL18BPa complex; free IL-18 was calculated (33).	The human myeloid data are correlative, the model leaves IL18BP-specific necessity unresolved, free IL-18 was calculated rather than measured directly, and the biomarker findings remain exploratory.
Renal cell carcinoma	Common-platform RNA and treatment-associated evidence: high *IL18R1*, *IL18RAP*, and *IL18BP* RNA in clear cell renal cell carcinoma, patient IL18BP protein dynamics, and exploratory serum composite biomarkers (11, 51).	RNA is not secreted protein or free IL-18; treatment-associated dynamics and composite scores do not validate standalone IL18BP use.
Glioblastoma	Time-resolved single-cell analysis in a mouse glioblastoma model showed increased *Il18bp* transcript within a suppressive monocyte-to-macrophage trajectory (49).	The transcript trajectory alone does not establish IL18BP-specific induction, secreted protein, neutralizing activity, or causality.
Pancreatic cancer/pancreatic ductal adenocarcinoma	Tumor-derived IL-18 supported regulatory-B-cell-associated tolerance in a mouse pancreatic model; IL18BP-mediated IL-18 neutralization combined with PD-1/PD-L1-pathway inhibition reduced tumor growth and metastasis (12). IL-18 receptor signaling was linked to tumor-reactive CD8-positive T-cell exhaustion in separate murine models (35).	The models involve different responders and do not establish native IL18BP baselines or induction mechanisms across pancreatic cancers.
Hepatic metastasis/melanoma	IL-18 promoted hepatic endothelial adhesion and metastatic retention; IL18BP reduced these readouts in mouse B16 melanoma models (13, 14).	This older intervention evidence is specific to B16 mouse hepatic-metastasis models and cannot define patient baselines or generalize across hepatic metastatic settings.
Colorectal liver metastasis	Interferon-γ induced IL18BP secretion in selected MMR-d patient-derived tumor organoids. Normal-liver-derived γδ cells showed IL-18 responsiveness; IL18BP blockade enhanced IL-18-driven responses of PBMC-derived, expanded Vδ1-positive T cells in separate organoid co-cultures (50).	These ex vivo and organoid findings are context-specific and do not establish patient treatment benefit.
Lymphoma/acute myeloid leukemia	Early human studies tested IL-18-armored cellular therapies in lymphoma and acute myeloid leukemia (44, 45).	These clinical platform results do not establish endogenous disease IL18BP baselines or induction, or the susceptibility of the secreted ligand to IL18BP.

CD, cluster of differentiation; IL, interleukin; IL18BP, interleukin-18-binding protein; *IL18R1*, IL-18 receptor alpha-chain gene; *IL18RAP*, IL-18 receptor accessory-protein/beta-chain gene; TP53, tumor protein p53; TREM2, triggering receptor expressed on myeloid cells 2. MMR-d, mismatch repair-deficient; PBMC, peripheral blood mononuclear cell; PD-1, programmed cell death protein 1; PD-L1, programmed death ligand 1; Vδ1, T-cell receptor delta variable 1.

Quantitative cross-cancer ranking of baseline IL-18 or IL18BP is not possible from the available studies. They sample different compartments-including tumor tissue, ascites, blood, urine, cell culture, organoids, and mouse tumors-and measure non-equivalent analytes such as RNA, total protein, IL-18/IL18BP complexes, expression ratios, calculated free IL-18, or functional neutralization. Shared pan-tumor transcriptomic analyses provide a limited common-platform comparison of *IL18BP* RNA and place clear cell renal cell carcinoma among tumor types with high *IL18R1*, *IL18RAP*, and *IL18BP* RNA (10, 11). These RNA data cannot be translated into secreted IL18BP protein or receptor-competent free IL-18, and other comparisons remain meaningful only within their original study and assay framework.

Direct induction has been tested in only a subset of models. IFN-γ induced functional IL18BP in selected prostate cancer cell lines; IFN-γ and IL-27 induced IL18BP in ovarian cancer models; and IFN-γ increased IL18BP secretion in colorectal patient-derived tumor organoids (29, 31, 50). In lung cancer and glioblastoma, *Il18bp* transcripts were associated with defined myeloid programs. Tumor-debris uptake promoted the TREM2-associated program in the lung model; these program-level findings do not by themselves isolate an IL18BP-specific induction mechanism (48, 49).

### 4.1 Direct tumor IL18BP source evidence: ovarian and prostate cancer

The ovarian and prostate studies summarized in Section 3 identify cytokine-responsive tumor-cell secretion (29, 31). Tissue expression ratios, macrophage staining and biofluid detectability provide complementary evidence of expression balance, localization and association (29, 34).

### 4.2 Lung cancer, renal cell carcinoma, gastric cancer, and glioblastoma: associations and treatment context

The macrophage-NK circuit described in Section 3 was tested mainly in mouse lung tumors, with human NSCLC data providing correlative context (48). A separate metastatic NSCLC cohort provides an exploratory plasma biomarker example, discussed in Section 7 (33).

Renal cell carcinoma provides common-platform RNA, treatment-associated, biomarker, and preclinical intervention evidence. Pan-tumor analyses place clear cell renal cell carcinoma among contexts with high *IL18R1*, *IL18RAP*, and *IL18BP* RNA, and small patient studies report treatment-associated IL18BP protein dynamics (11). A separate retrospective analysis included IL-18 and IL18BP within multianalyte composite biomarker scores (51). Within this mixed evidence base, standalone IL18BP remains unvalidated as a biomarker, and the mouse combination results cannot define a general checkpoint-combination rule.

In gastric adenocarcinoma, Huang et al. linked *IL18BP* promoter accessibility and expression with *TP53* and CD4-positive T-cell infiltration in a correlative multi-omics and tissue-validation study (52).

The glioblastoma study described in Section 3 tracked *Il18bp* expression as monocytes developed into suppressive macrophages in mouse tumors (49).

### 4.3 Pancreatic cancer and hepatic metastatic niches: divergent roles of IL-18 and IL18BP

Pancreatic-cancer models identify suppressive effects in different responder compartments. Tumor-derived IL-18 supported regulatory-B-cell-associated tolerance and impaired NK- and cytotoxic-T-cell activity in one model, in which IL-18 neutralization with IL18BP combined with programmed cell death protein 1/programmed death ligand 1 pathway inhibition reduced tumor growth and metastasis (12). A separate pancreatic model linked IL-18 receptor signaling in tumor-reactive CD8-positive T cells to an exhaustion program (35). Because the studies probe different responder compartments, they do not provide a comparable native IL18BP baseline or endogenous induction program across pancreatic cancers.

Hepatic metastatic niches illustrate opposing implications. In mouse B16 melanoma models, IL-18 promoted hepatic endothelial adhesion and metastatic retention, whereas IL18BP intervention reduced these readouts (13, 14). A study of colorectal liver metastasis in a mismatch repair-deficient/microsatellite-unstable context found IL-18-responsive gammadelta T (γδ T) cells in ex vivo assays using normal-liver-derived cells. In separate organoid co-cultures, cancer-cell IL18BP restrained IL-18-driven activity of PBMC-derived, expanded Vδ1-positive T cells, and IL18BP blockade enhanced their responses. IFN-γ also increased IL18BP secretion in selected patient-derived tumor organoids (50). The opposing results arise from distinct diseases and model systems and cannot support a uniform choice between IL18BP blockade and augmentation in hepatic metastasis.

### 4.4 Hematologic malignancies: platform-based clinical evidence

In lymphoma and acute myeloid leukemia, the evidence considered here comes primarily from early human testing of IL-18-armored cellular therapies (44, 45), rather than from direct characterization of endogenous IL18BP regulation. These clinical platform results do not establish endogenous IL18BP regulation, its importance as a native checkpoint, or the secreted ligand’s resistance to IL18BP.

Across cancer types, source mapping, functional neutralization, biomarker development, and treatment hypotheses require different forms of evidence. The studies compared here do not establish a clinical rule for choosing between IL18BP blockade and IL-18 neutralization.

## 5 Dual roles of IL-18 signaling in cancer

As summarized in **Figures 2A** IL-18 is neither constitutively antitumor nor constitutively protumor. Its direction depends on the amount of mature free ligand, receptor competence, the responding cell, the available co-signal, molecular form, exposure schedule, tissue niche, and treatment background. IL18BP can therefore be a therapeutic barrier when it sequesters IL-18 needed by antitumor lymphocytes, or a protective buffer when free IL-18 feeds suppressive, endothelial, or tumor-cell circuits.

### 5.1 Antitumor IL-18 signaling: NK cells, CD8-positive T cells, and IFN-γ-linked immunity

Receptor-proximal signaling follows the sequential IL-18Rα/IL-18Rβ and MyD88-IRAK-TRAF6 architecture described above, whereas downstream branches and functional outputs vary with the responder and co-signal. In the human NK-92 cell line, IL-18 activated signal transducer and activator of transcription 3 (STAT3) and ERK1/2; with trace interleukin-2 (IL-2), ERK- or p38-pathway inhibition reduced IFN-γ production and K562 cytolysis, whereas IL-18 alone induced *IFNG* mRNA without detectable protein (26). The effector requirements differed in murine pulmonary-metastasis models: IL-18 monotherapy required tumor sensitivity to Fas ligand (FasL), whereas IL-2 plus IL-18 required NK cells and engaged both FasL and perforin (53).

IL18BP-resistant agonism provides tumor-model evidence for both T-cell and NK-cell outputs. In immunogenic mouse tumors, decoy-resistant IL-18 (DR-18) required IL18R signaling, CD8-positive T cells, and IFN-γ; it expanded T-cell factor 1 (TCF1)-positive precursor cells, reduced thymocyte selection-associated high mobility group box (TOX)-high exhausted states, and increased polyfunctional CD8-positive T-cell and NK-cell readouts, including IFN-γ, granzyme B, and CD107a (10). The effector requirements differed in major histocompatibility complex class I (MHC-I)-deficient tumors: NK1.1-positive cells and IFN-γ were required for DR-18 activity, whereas FasL blockade or perforin deficiency did not abrogate DR-18 tumor control in the MC38 *B2m*-deficient model (10).

Anti-IL18BP blockade releases endogenous ligand rather than supplying an engineered agonist. COM503 displaced IL-18 from preformed IL18BP: IL-18 complexes and restored IFN-γ and tumor necrosis factor alpha (TNF-α) production by human CD8-positive tumor-infiltrating lymphocytes and IFN-γ production by IL-12-conditioned human NK cells (9). In mouse tumors, surrogate blockade increased polyfunctional effector T and NK states. In the MC38-OVAdim model, depletion of CD8-positive T cells or NK cells removed antitumor activity. The poorly infiltrated 4T1 and LLC models were resistant to this treatment (9).

In renal cell carcinoma (RCC) models, DR-18 paired more effectively with anti-cytotoxic T-lymphocyte-associated protein 4 (CTLA-4) than with anti-programmed cell death protein 1 (PD-1). In the Renca model, the DR-18/anti-CTLA-4 combination depended on CD8-positive T cells, NK cells, and IFN-γ (11). These preclinical results depend on competent responder cells and treatment context. Anti-IL18BP releases an endogenous ligand reservoir, whereas DR-18 supplies an agonist that evades the decoy (9, 10).

### 5.2 Protumor or immunosuppressive IL-18 signaling

Suppressive outcomes also begin in defined responder cells. In pancreatic-cancer systems, tumor-derived IL-18 promoted IL18R-positive regulatory B-cell expansion, interleukin-10 (IL-10) production, checkpoint-associated immune tolerance, and reduced NK- and cytotoxic-T-cell killing; IL-18 neutralization with IL18BP, combined with inhibition of the PD-1/programmed death ligand 1 (PD-L1; also B7-H1) pathway, reduced tumor and metastasis readouts in the tested mouse model (12).

In a murine PancOVA model, *Il18r*-deficient tumor-reactive CD8-positive cytotoxic T lymphocytes (CTLs) showed greater tumor necrosis factor/IFN-γ function and lower coinhibitory-receptor, eomesodermin, and TOX readouts. Lower coinhibitory-receptor and TOX expression was also observed in an orthotopic KPC model. An *in vitro* exhaustion assay linked the IL18R-dependent program to IL-2/signal transducer and activator of transcription 5 (STAT5) and protein kinase B (AKT)-mechanistic target of rapamycin (mTOR) signaling (35). The Zhao and Lutz studies thus implicate different responding compartments-regulatory B cells and tumor-reactive CD8-positive T cells, respectively-rather than a shared, uniformly protumor IL-18 program.

IL-18 can also redirect NK-cell differentiation. In B16F10 and CT26 mouse metastasis models, tumor-derived or intermittently administered IL-18 promoted Kit-positive CD11b-negative NK cells with high PD-L1. Host IL18R/MyD88 was required for their accumulation in the B16F10 model. Adoptive transfer of these cells promoted metastasis and reduced dendritic-cell and mature-NK-cell pools in a PD-L1-dependent manner, whereas tumor IL-18 knockdown or systemic IL18BP reduced their accumulation (36, 37). The same study series reported opposing outcomes for intermittent and daily IL-18 regimens, indicating that the dose and exposure schedule can change the functional direction observed in these models.

Non-lymphoid responders provide additional protumor routes. In murine hepatic-metastasis models, IL-18 participated in an endothelial program linked to interleukin-1 beta (IL-1β) and tumor necrosis factor that increased vascular cell adhesion molecule 1 (VCAM-1)-dependent B16 melanoma adhesion; IL18BP reduced VCAM-1 up-regulation, early hepatic retention, and metastatic burden (13, 14).

In human liver-cancer cells, IL-18 induced ERK2-dependent phosphorylation of the tumor-cell adaptor acyl-CoA synthetase long-chain family member 6 (ACSL6), stabilized the IL18R1-IL18RAP complex, and strengthened TRAF6/transforming growth factor-beta-activated kinase 1 (TAK1)-NF-κB signaling. In mouse tumor models, the resulting C-X-C motif chemokine ligands 1 and 5 (CXCL1/CXCL5) program recruited neutrophils and macrophages and reduced cytotoxic CD8-positive T-cell infiltration (38). These studies place the relevant IL-18 responses in hepatic endothelium and IL18R-competent liver-cancer cells, respectively, leaving the implications for neutralization dependent on the responding compartment.

The molecular form of IL-18 also affects its activity. In gallbladder-cancer models, polypyrimidine tract-binding protein 3-mediated exon skipping generated a ΔIL-18 isoform that reduced F-box only protein 38 transcription, stabilized PD-1 in CD8-positive T cells, and promoted immune escape, whereas classic IL-18 enhanced CD8-positive T-cell activity in the same study (39). These opposing activities distinguish the tumor-associated splice isoform from classic IL-18; these splice-form effects do not by themselves establish how ΔIL-18 engages IL-18R or is regulated by IL18BP, so they cannot be used to infer the effect of IL18BP blockade.

### 5.3 Contextual determinants and therapeutic interpretation

These contrasting effects of IL-18 depend on the biological context. Antitumor activity is most plausible when mature free IL-18 reaches receptor-competent NK cells or antigen-experienced T cells with suitable co-signals and elicits IFN-γ or model-defined cytotoxic mechanisms. Suppressive activity becomes plausible when the responding compartment is a regulatory B cell, an exhaustion-prone CD8-positive T cell, an immunoablative NK-cell precursor, hepatic endothelium, or an IL18R-competent tumor cell. Dose and schedule, acute versus sustained exposure, molecular form, and tissue localization further alter these routes.

Therapeutic interpretation must follow the measured axis state. Releasing endogenous IL-18 is supported by models in which IL18BP-bound ligand and responsive antitumor lymphocytes coexist; DR-18 instead supplies an IL18BP-resistant agonist to engage responsive cells (9–11). Preserving or augmenting neutralization remains plausible when IL-18 drives B-regulatory tolerance, immunoablative NK states, endothelial adhesion, or tumor-cell-driven myeloid recruitment with reduced CD8-positive T-cell infiltration (12–14, 36–38). These cited model experiments support hypotheses about axis state without supplying validated clinical markers for choosing between blockade and neutralization; early human development is discussed in Section 8.

## 6 IL18BP as a soluble checkpoint-like regulator and therapeutic barrier

The checkpoint-like role of IL18BP is functional: IL18BP acts extracellularly and differs structurally from membrane checkpoints such as PD-1 or CTLA-4. IL18BP is a secreted antagonist that can sequester extracellular mature IL-18 before sequential engagement of IL-18Rα and IL-18Rβ (5, 6). It becomes a checkpoint-like barrier only when the restrained receptor-competent ligand would otherwise reach antitumor responder cells. Its abundance is therefore interpretable only alongside ligand occupancy, functional neutralization, and responder context. IFN-γ can reinforce this extracellular barrier by inducing IL18BP in selected epithelial and carcinoma-compatible systems (7, 8).

Preclinical studies test two mechanistically distinct ways to overcome this barrier. Anti-IL18BP blockade increases endogenous ligand availability, whereas DR-18 and verified resistant variants supply an agonist that bypasses sequestration (9, 10). These approaches and their early human development are discussed in Section 8.

In selected pancreatic or hepatic-metastasis models, IL-18 instead supported suppressive or metastatic circuits, so preserving or increasing neutralization remained protective in those experimental settings (12–14). These contrasting models make ligand availability and responder context central to interpreting the checkpoint-like analogy.

## 7 Biomarker potential

The IL-18/IL18BP axis should be evaluated as a set of distinct measurement domains rather than as a single-marker test. At minimum, research studies need to separate ligand partitioning, antagonist abundance, responder-cell mapping, tissue source, and treatment-linked change. **Table 1** organizes these domains as research readouts; combining them into a clinical panel or decision tool will require prospective validation.

Tissue or bulk IL18BP expression can help locate a source or cellular state; evidence of secreted functional protein and IL-18 neutralization requires complementary assays (29, 31, 34, 48, 49). Spatial profiling, single-cell transcript analysis, immunohistochemistry, and matched protein measurements are complementary, with each resolving a different part of the source-to-function relationship.

Soluble analytes answer a different question. Total IL-18 reports assay-detectable ligand abundance without resolving its bound, free, or biologically inactive fractions, whereas IL18BP concentration alone leaves ligand occupancy unknown. In the metastatic non-small cell lung cancer study by Janho Dit Hreich et al., total IL-18, IL18BPa, and IL-18/IL18BPa complex were measured, whereas free IL-18 was calculated from measured analytes and binding assumptions rather than directly measured as a standalone analyte (33). Likewise, an IL-18/IL18BP ratio describes axis imbalance without directly measuring free IL-18 (34). Each report should state explicitly whether free IL-18 was directly measured or mathematically estimated and which assumptions were used (32, 33).

Existing oncology associations remain exploratory. IL18BP has been detected in prostate-cancer-associated biofluids (29). In metastatic renal cell carcinoma, Lee et al. analyzed individual IL18BP associations and incorporated IL18BP and IL-18 into multianalyte models (51). The human cohorts studied by Schoenfeld et al. relate receptor and IL18BP context to treatment background within renal cell carcinoma (11). These studies motivate prospective testing; cutoffs, survival prediction, and treatment assignment require separate validation.

Future trials can use longitudinal sampling to ask whether an intervention changes ligand partitioning, IL18BP abundance, responder-cell state, and inflammatory safety signals (33, 54).

## 8 Therapeutic strategies targeting the IL-18/IL18BP axis

Therapeutic development has moved from systemic native IL-18 toward control of extracellular availability, molecular form, location, duration, and responder targeting. Systemic recombinant IL-18 remains the historical baseline; newer strategies release endogenous IL18BP-bound ligand, bypass sequestration with verified resistant agonists, localize or temporally control expression, target or bridge receptor activation, or increase neutralization in selected suppressive contexts. **Figure 2B** summarizes five intervention strategies. **Table 3** compares these strategy classes across preclinical and early clinical research by localization and temporal control, relationship to IL18BP, evidence stage, and main trade-off.

**Table 3:** Delivery, IL18BP susceptibility, and evidence for therapeutic strategies.

Strategy	Delivery and duration	Relation to IL18BP	Evidence in cited studies	Main limitation or trade-off
Legacy recombinant IL-18	Intravenous cytokine; exposure depends on administration and is not tumor-localized (55).	Native IL-18 remains susceptible to IL18BP (5, 6, 10).	Phase I clinical precedent.	Historical baseline; it does not solve decoy sequestration or tumor localization.
Anti-IL18BP blockade	No new IL-18 delivered; functional localization depends on coexisting endogenous mature IL-18 and responsive cells (9).	COM503 and tested surrogates block IL18BP and can release precomplexed ligand.	Human tumor and ex vivo mechanistic evidence; mouse antitumor efficacy using surrogate antibodies (9); subsequent COM503 phase 1 study (NCT06759649).	Depends on endogenous mature IL-18 availability and an appropriate antitumor responder context.
Engineered IL-18 proteins, including DR-18	Soluble proteins lack inherent tumor localization; persistence varies by construct, including Fc-based half-life extension (63, 64).	DR-18 and Dreaden variants evade IL18BP, whereas the Bainbridge IL-18-Fc construct retains IL18BP sensitivity (10, 11, 63, 64).	Preclinical evidence for the cited constructs; the related engineered IL-18 agonist ST-067 has entered early human testing (NCT04787042).	Decoy resistance, stability, and persistence vary by construct; IL18BP evasion also removes a physiological brake.
Local mRNA delivery	Intratumoral mRNA or transiently mRNA-engineered cells; transient expression may require repeat delivery (40, 41, 59).	DR-18 payloads evade IL18BP (40, 41); the multiplexed mouse-IL-18 cocktail results do not establish IL18BP resistance (59).	Preclinical.	Route, formulation, co-payloads, and innate sensing complicate IL-18-specific attribution.
Viral delivery of IL18BP-resistant IL-18	Tumor-directed or oncolytic expression; localization and duration depend on vector, route, tropism, and replication (42, 56–58).	Representative studies encode IL18BP-resistant IL-18 variants.	Preclinical.	Vector, oncolysis, innate sensing, and co-payload effects limit cross-platform comparison and IL-18-specific attribution.
IL-18-armored cell therapies	Production site and duration depend on engineered-cell trafficking, expansion and persistence; secreted cytokine exposure need not remain confined to those cells (43–45).	Native secreted IL-18 remains susceptible unless specifically engineered for resistance.	Early clinical evidence in lymphoma/AML plus preclinical evidence.	Persistent cellular exposure, inflammatory safety, and solid-tumor generalizability require platform-specific evaluation.
Targeted and synthetic agonists	Target-cell- or receptor-directed; not necessarily tumor-localized. Persistence varies by construct (46, 47, 61, 62).	Individual formats may evade IL18BP or bypass the ligand by receptor bridging.	Preclinical or engineering/method stage.	Human selectivity and safety await evaluation across formats with different mechanisms and maturity.
IL-18 neutralization	Neutralizes IL-18 rather than delivering an agonist (12–14).	Adds or mimics IL18BP function.	Preclinical, model-specific evidence.	May be protective in suppressive or metastatic niches but could blunt useful antitumor IL-18 signaling elsewhere.

IL-18, interleukin-18; IL18BP, interleukin-18-binding protein; DR-18, decoy-resistant IL-18; mRNA, messenger RNA; Fc, fragment crystallizable region; AML, acute myeloid leukemia.

An early recombinant wild-type IL-18 trial provides clinical precedent for IL-18 agonism (55). Yet native IL-18 remains vulnerable to IL18BP sequestration, so systemic cytokine administration should not be assumed to create durable receptor-competent free IL-18 inside an IL18BP-rich tumor microenvironment (5, 6, 10). It also does not solve localization, half-life, patient selection, or inflammatory safety.

### 8.1 Releasing endogenous IL-18 or bypassing IL18BP

Anti-IL18BP blockade aims to increase endogenous mature IL-18 availability to responsive antitumor cells. A recoverable IL18BP-bound pool can provide ligand for release. Menachem et al. provide preclinical support for this mechanism, including evidence for IL18BP-bound IL-18 in tumor samples, interference with IL18BP: IL-18 complexes, functional T and NK cell assays, and surrogate anti-mouse IL18BP antitumor activity in mouse models (9). COM503 has subsequently entered a phase 1 study in advanced solid tumors, assessing safety, tolerability, and dose selection (NCT06759649). The rationale weakens when IL-18 signaling supports tolerance, metastatic niche formation, or systemic inflammation.

Decoy-resistant IL-18 variants provide an alternative way to overcome sequestration. They do not remove IL18BP; they replace the ligand with an agonist designed to signal through IL-18R while evading the soluble decoy. Zhou et al. present DR-18 as an IL18BP-resistant IL-18 strategy with preclinical CD8 and NK immune-remodeling activity (10). Schoenfeld et al. examined DR-18 in an RCC-specific treatment context involving tumor lineage and checkpoint background (11). The engineered IL-18 agonist ST-067 has entered early human testing in a study designed with phase 1a dose escalation and phase 2 expansion; that design does not establish that the expansion has enrolled or that clinical efficacy has been demonstrated (NCT04787042).

### 8.2 Delivery platforms and targeted receptor activation

mRNA and viral approaches attempt to focus IL-18 or DR-18 activity in tumor tissue, engineered immune cells, or oncolytic compartments rather than across the whole circulation. Examples include intratumoral or transient mRNA delivery, multiplexed cytokine or antigen mRNA programs, adenoviral vectors, and oncolytic viral platforms (40–42, 56–59). Because IL-12, chemokines, antigen mRNA, interferon signals, oncolysis, vector tropism, and innate sensing can all shape the immune outcome, their effects cannot be attributed to IL-18 alone. The cytokine-expression and antitumor results of the multiplexed pancreatic mRNA study do not establish that its delivered IL-18 resists IL18BP (59).

IL-18-armored cells couple cytokine production to adoptively transferred immune cells. Early human studies have tested IL-18-armored cellular platforms in hematologic malignancies (44, 45). Preclinical studies and secondary syntheses help explain the rationale that IL-18 secretion could support expansion, persistence, and remodeling of suppressive compartments, while remaining separate from solid-tumor IL18BP-axis claims (43, 60). Cellular delivery does not by itself establish escape from IL18BP; receptor-accessible ligand depends on the secreted construct, local production, and neutralization in the relevant compartment. Constitutive secretion also raises safety questions, making inducible, tumor-gated, or transient designs important future directions.

PD-1-directed attenuated IL-18, bispecific mimetics, surrogate agonists, and receptor-bridging approaches all attempt to control the target cell, receptor geometry, or IL18BP susceptibility of the signal (46, 47, 61, 62). These strategies shift the design question from “how much IL-18 is present” to “which receptor-bearing cell receives what kind of IL-18R signal.” Much of this evidence remains preclinical or engineering-stage, so these formats remain emerging directions rather than mature therapeutic categories.

### 8.3 IL-18 neutralization in tumor-supportive settings

IL-18 neutralization remains relevant when IL-18 is tumor-supportive. In the pancreatic model studied by Zhao et al., tumor-derived IL-18 promoted regulatory B-cell tolerance through the PD-1/PD-L1 pathway; IL-18 neutralization with IL18BP combined with PD-1/PD-L1-pathway inhibition reduced tumor growth and metastasis (12). Hepatic metastasis studies support the idea that IL-18 can contribute to endothelial adhesion and metastasis-related biology, and that IL18BP intervention can be protective in selected models (13, 14). 

### 8.4 Comparing localization, duration, and IL18BP susceptibility

These formats address different delivery and control problems. Soluble engineered IL-18 proteins lack intrinsic tumor localization, while their persistence and IL18BP susceptibility depend on the construct: DR-18 and some fragment crystallizable (Fc)-fused variants evade IL18BP, whereas a stabilized half-life-extended IL-18-Fc retained IL18BP sensitivity (10, 11, 63, 64). Intratumoral or cell-associated mRNA can provide transient expression, whereas viral expression depends on vector, route, tropism, and replication (40–42, 56–59). In armored-cell platforms, the site and duration of cytokine production depend on cell trafficking and persistence; these properties do not by themselves establish tumor-confined exposure or escape of native IL-18 from IL18BP (43–45). Targeted and receptor-bridging agonists pursue cell or receptor selectivity, which does not by itself establish tumor-confined delivery (46, 47, 61, 62).

Across strategy classes, translational studies should distinguish changes in total, bound, directly measured free, and calculated free IL-18, while resolving where and for how long signaling occurs, which responder cells are engaged, and whether systemic inflammatory circuits are activated. These measures provide a comparative research framework whose role in patient selection will depend on prospective validation.

## 9 Challenges and future perspectives

Three linked gaps now limit translation of the IL-18/IL18BP axis: resolving ligand state, identifying the producing and responding cells, and controlling where and for how long an intervention changes signaling. Future studies should state the sampling compartment and measurement method and should combine soluble analytes with spatial, cellular, protein, and functional source mapping where feasible (29, 31, 34, 48, 49). **Table 1** summarizes these domains for research use.

Prospective studies can connect these measurements to treatment by predefining the biological hypothesis being tested: whether mature ligand is present and how it is partitioned, which cells can respond, whether the expected response is antitumor or suppressive in the selected model, how the intervention changes localization or duration, and which inflammatory signals will be monitored. These intervention hypotheses require prospective testing, and their clinical decision-making value remains unvalidated (9–14).

The evidence behind these strategies spans distinct stages. Recombinant IL-18 provides historical clinical exposure, and IL-18-armored cells have entered early human testing in lymphoma and acute myeloid leukemia (44, 45, 55). Early human development of IL18BP blockade and engineered IL-18 agonism is described in Section 8. The evidence stage of each strategy is identified separately in **Table 3**. Human tissue evidence, mouse intervention, transcriptomic association, and engineered-platform studies each support different inferences, precluding their placement in a single efficacy hierarchy.

Safety evaluation should follow mechanism rather than platform labels. Evading IL18BP removes a physiological brake; local administration may still permit extra-tumoral signaling, target-cell engagement may occur without tumor localization, and cell-based expression introduces platform-specific persistence. As summarized in **Table 3**, future studies should report location and duration of signaling, construct-specific IL18BP susceptibility, intended responder-cell engagement, and systemic inflammatory monitoring. 

The central question is therefore not whether IL18BP is uniformly beneficial or harmful. It is whether a defined tumor context contains extracellular mature IL-18, how much remains receptor competent, which cells can respond, and whether changing that balance can be controlled safely. This precision-tuning framework connects measurement, mechanism, and therapeutic design, while its clinical value remains to be tested (1, 9, 10, 15).
